# A national cross-sectional survey of constipation in patients attending cancer centres in Ireland

**DOI:** 10.12688/hrbopenres.13315.1

**Published:** 2021-10-15

**Authors:** Karen Ryan, Bridget M. Johnston, Clare McAleer, Laserina O'Connor, Philip Larkin

**Affiliations:** 1 University College Dublin, Dublin, D04 V1W8, Ireland; 2 St Francis Hospice Dublin, Dublin, D15 DE98, Ireland; 3 Department of Palliative Medicine, Mater Misericordiae University Hospital, Dublin, D07 AX57, Ireland; 4 Centre for Health Policy and Management, Trinity College Dublin, Dublin, D02 PN40, Ireland; 5 School of Nursing, Midwifery and Health Systems, University College Dublin, Dublin, D04 V1W8, Ireland; 6 Palliative and Supportive Care Service, Chair of Palliative Nursing, Lausanne University Hospital, Lausanne, CH-1011, Switzerland

**Keywords:** Constipation, neoplasms, guideline, palliative care, prevalence, cross-sectional studies

## Abstract

**Background: **The prevalence of constipation in patients with cancer is estimated at 50-90%. It is often associated with pain, anorexia, nausea and vomiting and impacts negatively on quality of life.

Despite its common occurrence, it is often poorly recognised and treated by healthcare professionals.

**Methods: **A national cross-sectional survey was conducted in Ireland to describe constipation prevalence and severity in patients attending cancer centres and to evaluate management efficacy.  In-patients or patients attending day oncology wards in any of the country’s eight designated cancer centres were eligible to participate. Participants were shown the Bristol Stool Chart  and answered questions regarding stool appearance and sensation of  incomplete defecation; they completed the Constipation Assessment Scale. Data on pain character and intensity, opioid use, and prescribed and over-the-counter laxative use were collected. Data were summarised using descriptive statistics. Significance of variations for continuous data were determined using t-tests. Conditional ordered logistic regression was undertaken to determine factors associated with constipation.

**Results:** The dataset comprised 491 patients. 24.8% had been reviewed by specialist palliative care; 14.5% by the anaesthetic pain team. In total, 42.2% of respondents were taking step 2 or step 3 opioids. Constipation prevalence was 67.6%; 19.4% of patients had Constipation Assessment Scale scores indicating severe constipation. A total of 46% of the respondents were not taking any laxatives. Of those who were taking laxatives, 54.8%  reported constipation symptoms. While opioid use was strongly associated with participants reporting higher scores, this association was not seen in those patients receiving specialist palliative care.

**Conclusions: **Constipation remains a clinical problem in Irish cancer centres. Despite increased opioid use, patients receiving specialist palliative care were more likely to take laxatives and reported less constipation. Specialist palliative care practice should be studied in order to identify what are the transferable ‘ingredients’ of effective constipation management.

## Introduction

Ireland has well-developed cancer and palliative care services and there is a growing focus on evidence-based practice and the development of learning healthcare systems ^1^. The management of cancer-related constipation has emerged as an area for improvement because it is a common condition that impacts negatively on quality of life, yet remains poorly recognised and treated ^2– 4^. Accordingly, a national clinical guideline focused on constipation management for patients receiving palliative care was published in 2015 ^5^.

This study was carried out to establish the prevalence of constipation in patients attending cancer centres and to assess the efficacy of treatment. Little is known of the extent of constipation burden experienced by patients with cancer in Ireland. Reliable information on management efficacy is lacking. The data will be of value as a source of comparative data given relative paucity of recent prevalence studies in cancer populations ^6– 8^. We hope to stimulate consideration of the factors associated with improved outcomes and re-double efforts to reduce constipation burden.

## Methods

### Ethics statement

Ethical approval was obtained in each cancer centre (Beaumont Hospital Research Ethics Committee – Ref: 17/48; Galway University Hospitals: C.A. 1739; HSE South-Eastern Area Research Ethics Committee - approval date: 24.05.17; Mater Misericordiae University Hospital Research Ethics Committee Ref: 1/378/1913; St James’s Hospital /AMNCH Research Ethics Committee Ref: 2017-05 Chairman’s Action (10); St Vincent’s Ethics and Medical Research Committee - approval date 19.04.17; University College Cork Clinical Research Ethics Committee – Ref: ECM 4 (m) 09/05/17; University Hospital Limerick, Research Ethics Committee Ref: 062/17) and the lead University (University College Dublin Research Ethics Committee LS-E-17-105-O’Connor; 16.6.17). Written, signed informed consent was obtained via signature from each participant for data collection (survey and chart review) and publication of results.

### Study design and subjects

A cross-sectional point prevalence study was carried out in the eight designated cancer centres in Ireland in 2017 (Beaumont Hospital, Galway University Hospital, University Hospital Waterford, Mater Misericordiae University Hospital, St James’s Hospital, St Vincent’s University Hospital, Cork University Hospital, University Hospital Limerick).

Pilot testing was initially carried out with a convenience sample of seven patients in one cancer centre. The pilot study provided a number of valuable logistical insights, such as providing an accurate estimation of time required to complete data collection, and how best to manage the distribution and storage of each of the three copies of the consent forms (patient, medical record and research team copies). Only minor changes to the data collection instrument were made, however. The term ‘medical chart’ was replaced with ‘medical record’ as the latter was better understood by participants and data collectors. Also, adopting the practice of Woolery *et al*., ^9^ statements using lay terminology were used as clarifying descriptors for each Constipation Assessment Scale (CAS) item for any participants who did not understand the original CAS item. 

Subsequently, data collection was carried out on a single day in each centre. It had been intended to carry out the study in all centres on the same day but operational issues meant that data was collected in two hospitals one week later.

A wide range of clinicians (consultants, specialist registrars, clinical nurse specialists, Advanced Nurse Practitioners, Assistant Directors of Nursing and Nurse Tutors) acted as data collectors for the study. All data collectors completed mandatory pre-study training comprising an e-learning presentation explaining how to follow the study protocol, carry out data collection and record data. A member of the research team was also present on the study day to oversee data collection and provide any additional support, as needed. 

In-patients or patients attending day oncology wards were eligible to participate. Inclusion criteria were: (1) cancer diagnosis; (2) informed of diagnosis (3) aged ≥18 years; (4) English speaking. Exclusion criteria were: (1) surgery ≤24 hours prior to the study; (2) cognitive impairment or reduced level of consciousness; (3) patient deemed too unwell to participate by the clinician. The inclusion criteria and data collection methods were applied consistently across all study sites to reduce the potential for bias.

### Data collection

Data collectors first liaised with clinical nurse managers of each ward in order to identify eligible patients and then approached the potential participant. The data collector provided both verbal and written information on the study to the patient and answered any questions that were asked. Following this, patients were invited to take part in the study on that day. Although a ‘cooling off’ period of one hour was offered, patients could waive that if desired. 

Demographic details were collected (gender and age) and the Constipation Assessment Scale (CAS) ^10^ completed ^11^. Respondents were shown the Bristol Stool Chart ^12^ and asked “Can you look at this scale and thinking of the last time you had your bowels open, which picture best resembles what it looked like?”. Two questions were asked about medications: ‘Are you currently taking medication prescribed by your doctor to manage your constipation?’ and ‘Are you currently taking medication that you purchased yourself to manage your constipation?’ Details on diagnosis, treatment and analgesics were extracted from chart review (see *Extended data* for a copy of the survey instrument ^13^).

### Analysis

Data were summarised using descriptive statistics. Significance of variations for continuous data were determined using t-tests.

Two outcomes were examined: 1) the Constipation Assessment Scale (CAS); and 2) laxative use.

1) Conditional ordered logistic regression was undertaken to determine factors associated with constipation. A categorical variable based on CAS scores was the dependent variable: no constipation (CAS score=0); mild constipation (CAS score 1–6) or severe constipation (CAS score 7–16). The independent variables were:

- Age - categorised during data collection in accordance with requirements for ethical approval at study sites.
- Gender- collected as a binary variable at the time of data collection.
- Primary cancer site - grouped into nine categories according to site of origin
- Currently receiving chemotherapy - binary variable.
- Currently receiving radiotherapy - binary variable.
- Currently using opioids - binary variable (Step 3 opioid use)
- Currently utilising specialist palliative care services – binary variable
- Laxative use - categorised into 4 groups capturing observed utilisation patterns: no use; using over-the counter only; using prescription only; using a combination prescription and over-the-counter laxatives.

2) Random effects logistic regression was undertaken to examine factors associated with laxative use. The binary dependent variable was laxative use (yes if taking any type of laxative). The independent variables were:

- Age, gender, receiving chemotherapy, receiving radiotherapy, utilising specialist palliative care services and opioid use - these were handled in the same manner as described above
- Additionally, the CAS score total was included as a continuous variable.

Analyses were carried out using Stata 15 ^14^, and tests of statistical significance were at *p*⩽0.05. In both models, odds ratios (OR) and 95% confidence intervals (CI) were estimated for each independent variable. All cases with incomplete data for the CAS items were excluded from regression analysis. Number of missing cases are shown in all relevant tables ( Table 3 and Table 4).

Reporting was provided according to the Strengthening the Reporting of Observational Studies in Epidemiology (STROBE) criteria ^15^ (see *Extended data* for a copy of the completed STROBE checklist ^13^).

## Results

### Participants

In total, 491 patients were recruited ( Table 1); 51.5% were female and 46.2% were aged ≥65 years. Haematological, breast and genitourinary cancers were the most common diagnoses. 44.6% were attending Day Oncology or Haematology services, with the remainder receiving inpatient care. The Specialist Palliative Care team were providing care to 24.8%; 14.5% had input from the Anaesthetic Pain Team. Pain was reported by 62.5% and Step 2 or 3 analgesics were being used by 42%. Patients who were receiving Palliative Care or Pain team input were more likely to be taking step 2 or 3 analgesics than patients who were not receiving input (p<0.001).

**Table 1.** Participant characteristics.

	Frequency	Percentage
**Gender:** Male Female Missing	206 253 32	42.0 51.5 6.5
**Age groups:** 18-39 40-64 65-79 80+ Missing	43 216 191 36 5	8.7 44.1 39.0 7.3 1.0
**Cancer Site:** Haematological Breast Genitourinary Lung Lower Gastrointestinal Upper Gastrointestinal Neurological Other/Multiple sites Missing	117 77 64 49 49 43 9 63 20	23.8 15.7 13 9.8 9.8 8.8 1.8 12.8 4
**Anti-tumour treatment:** Receiving chemotherapy Not receiving chemotherapy Missing Receiving radiotherapy Not receiving radiotherapy Missing Had surgical intervention Did not have surgical intervention Missing	351 123 17 176 296 19 241 234 16	71.5 25.0 3.5 35.8 60.3 3.9 49.1 47.7 3.3
**Laxative use:** None Prescribed laxatives Over-the-counter laxatives (self-medicating) Both prescribed and over-the-counter laxatives Missing/ not applicable (stoma)	226 158 22 39 46	46 32.2 4.5 8.0 9.3
**Specialist team involvement:** Specialist palliative care (‘SPC’) involved in care SPC not involved in care Missing Specialist pain team (‘Pain team’) involved in care Pain team not involved in care Missing	122 339 30 71 371 49	24.9 69.0 6.1 14.5 75.6 10.0
**Site of recruitment:** Cancer centre 1 Cancer centre 2 Cancer centre 3 Cancer centre 4 Cancer centre 5 Cancer centre 6 Cancer centre 7 Cancer centre 8	32 50 27 64 89 91 95 43	6.5 10.2 5.5 13.0 18.1 18.5 19.4 8.8

### Constipation Assessment Scale score

The prevalence of constipation among all participants was 67.6%, based on CAS criteria of a score of ≥1. Only 8.3% of respondents reported no symptoms while 19.4% of respondents scored ≥7, indicating severe constipation.

Most commonly reported symptoms were reduced bowel movements (44.8%), change in the amount of gas passed rectally (44.8%) and abdominal distension or bloating (43.4%). The symptoms affecting patients most severely were reduced bowel movements (14.7%), abdominal distension or bloating (12%) and rectal fullness or pressure (11.4%). Further detail is provided in Table 2.

**Table 2.** Constipation Assessment Scale scores.

Item	No problem	Some problem	Severe problem	Missing data *
**Abdominal distension or bloating**	268 (54.6%)	154 (31.4%)	59 (12%)	10 (2%)
**Change in the amount of gas passed rectally**	254 (51.7%)	168 (34.2%)	52 (10.6%)	17 (3.4%)
**Less frequent bowel movements**	250 (51.0%)	148 (30.1%)	72 (14.7%)	21 (4.2%)
**Oozing liquid stool**	349 (71.1%)	86 (17.5%)	33 (6.7%)	23 (4.7%)
**Rectal fullness or pressure**	312 (63.5%)	103 (21.0%)	56 (11.4%)	20 (4.1%)
**Rectal pain with bowel movement**	343 (69.9%)	85 (17.3%)	42 (8.6%)	21 (4.3%)
**Smaller stool size**	288 (58.7%)	134 (27.3%)	40 (8.2%)	29 (6.0%)
**Urge, but inability to pass stool**	287 (58.5%)	131 (26.7%)	55 (11.2%)	18 (3.6%)

*Missing data or not collected

### Bristol Stool Chart

20.9% reported hard stools, 55.2% reported normal stools and 17.6% reported loose stools. A weak negative correlation (r =-0.1), was observed for CAS scores and stool type, indicating that lower CAS scores were associated with looser stool.

### Laxative use

Just under half (46%) were not taking laxatives. 32.2% were taking prescribed laxatives. Some patients were self-medicating by taking over-the-counter laxatives- 4.5% were taking over-the-counter laxatives alone, while a further 8.0% were taking both prescribed laxatives and supplementary over-the-counter laxatives. Despite taking laxatives, 54.8% of participants reported symptoms.

### Factors associated with constipation burden

Ordered logistic regression analysis was conducted to examine factors associated with constipation ( Table 3). Female gender was associated with increased odds of reporting constipation burden (OR 1.975, 95% CI: 1.16-3.35; *p*= 0.012). Those aged 80 and over were less likely to be constipated than those aged 18–39 (OR .285, 95% CI: .082-.995; *p*=0.49). Neither treatment with chemotherapy nor radiotherapy were associated with higher CAS scores. Unsurprisingly, opioid use was strongly associated with higher CAS scores (OR 2.19, 95% CI:1.30-3.67; *p*=0.003). Importantly, no association between receiving SPC and increased constipation burden was noted ( *p*=0.35). Patients who were constipated were more likely to be taking prescribed (OR 6.446, 95% CI: 3.43-12.15; *p*<.001), over-the-counter laxatives (OR 3.171, 95% CI: 1.064-9.450; *p*=0.04), or a combination of both (OR 21.957, 95% CI: 8.001-60.254; *p*<0.001).

**Table 3.** Ordered logistic regression examining factors associated with constipation burden.

Variable	Odds ratio	p-value	95% CI	
**Age category**				
***18–39***	REF			
***40–64***	.491	0.103	.209	1.155
***65–79***	.455	0.082	.187	1.103
***>79***	**.285**	**0.049**	**.082**	**.995**
**Cancer site**				
***Upper gastrointestinal***	REF			
***Lower gastrointestinal***	.698	0.552	.213	2.286
***Genitourinary***	.430	0.130	.144	1.281
***Neurological***	.275	0.237	.032	2.338
***Haematological***	**.376**	**0.050**	**.141**	**1.00**
***Breast***	.456	0.147	.158	1.318
***Lung***	**.281**	**0.029**	**.089**	**.880**
***Other***	.387	0.110	.121	1.240
***Multiple***	.347	0.185	.072	1.65
**Gender**				
***Male***	REF			
***Female***	**1.975**	**0.012**	**1.164**	**3.351**
**Receiving chemotherapy**	.786	0.445	.424	1.457
**Receiving radiotherapy**	1.055	0.836	.636	1.751
**Using opioids**	**2.189**	**0.003**	**1.304**	**3.672**
**Utilising specialist palliative care services**	1.33	0.352	.728	2.440
**Using laxatives**				
***Nothing***	REF			
***Prescribed***	**6.446**	**<0.001**	**3.430**	**12.114**
***Over-the-counter***	**3.171**	**0.038**	**1.064**	**9.450**
***Prescribed and over-the-counter***	**21.958**	**<0.001**	**8.001**	**60.254**

**Table 4.** Random effects logistic regression examining factors associated with laxative use.

Variable	Odds ratio	p-value	95% CI	
**Age category**				
***18–39***	REF			
***40–64***	.935	0.895	.344	2.543
***65–79***	2.149	0.140	.779	5.932
***>79***	1.098	0.899	.259	4.665
**Gender**				
***Male***	REF			
***Female***	.734	0.280	.418	1.287
**Receiving chemotherapy**	.759	0.426	.386	1.495
**Receiving radiotherapy**	.872	0.641	.489	1.552
**Utilising specialist palliative care services**	**2.953**	**0.002**	**1.476**	**5.905**
**Using opioids**	**1.824**	**0.042**	**1.022**	**3.257**
**CAS score**	**1.51**	**<0.001**	**1.354**	**1.685**

### Factors associated with laxative use

Although 39.8% experienced symptoms and were not taking laxatives, there was evidence that increased CAS scores were associated with increased odds of using laxatives (OR 1.510, 95% CI: 1.354-1.685; *p*<0.001). Being known to SPC services (OR 2.952, 95% CI:1.476-5.905; p=0.002) and opioid use (OR 1.824, 95% CI: 1.022-3.256; *p*=0.042) were also associated with increased odds of using laxatives.

## Discussion

Patients attending cancer centres in Ireland have a significant constipation symptom burden echoing other study findings and demonstrating little change over time ^6– 8^. Symptom profile was similar to the original CAS validation study ^10^. Female gender was associated with constipation, but opioid use, unsurprisingly, was most strongly associated.

A total of 39.8% of participants were not taking laxatives despite symptoms, and 54.8% remained constipated despite taking laxatives. This points to a need not only to encourage appropriate laxative use but also to improve prescribing. Patients known to Palliative Care might be expected to be at higher risk of constipation due to opioid use and complex symptomatology. However, an association between Palliative Care input and increased constipation burden was not observed. It is hypothesised that this is attributable to the fact that patients receiving Palliative Care benefitted from comprehensive symptom assessment and appropriate prescribing supported by guideline use. 

Different countries have taken different approaches to the development of constipation guidelines ^16^. In Ireland, the only national guideline on constipation is titled ‘Management of Constipation of Adult Patients Receiving Palliative Care’ ^5^. Acknowledging that misperceptions exist regarding the term ‘palliative care’ ^17^, and noting the high prevalence of constipation found in the broader cancer population studied, it is possible that the title acted as a barrier to uptake. Planning is key to successful guideline development ^18^, and identification of scope a critical first step ^19^. While it is possible to develop broad guidelines, considerable resources are required and influence decisions regarding scope. Given the lack of improvement seen to date, we suggest that a broader approach in future guidelines should be considered to reduce fragmentation of practice and build momentum in quality improvement.

Study limitations include the fact that patient experience outside cancer centres is not described. While the multi-site design adds to the robust nature of data collection, logistical challenges meant that the study was not carried out on a single day and centres demonstrated variability in recruitment. Finally, given the disparity of a single agreed definition for constipation, a potential confounder is chronic constipation as defined by ROME Diagnostic Criteria ^20^. Future studies should aim to characterise patients who have a pre-morbid diagnosis of chronic constipation.

## Conclusion

Cancer-related constipation remains inadequately recognised and treated. The merits of symptom assessment and guideline application as evidenced by lower symptom burden associated with Palliative Care input are suggested. The confirmation of the high prevalence of constipation in the wider population, reaffirms the need to find more effective approaches to quality improvement across the cancer trajectory.

## Data availability

### Underlying data

Due to the nature of this research and the consent document, participants of this study were not asked to consent to the sharing of data beyond the research team and their collaborators. As a result, underlying data cannot be publicly provided. Researchers seeking to access the underlying dataset will need to apply directly to all University and Hospital Research Ethics Committees for approval. UCD Office of Research Ethics can be contacted at research.ethics@ucd.ie; the individual hospital research ethics committees can be contacted at beaumontethics@rcsi.com; colette.collins@hse.ie; caroline.lamb2@hse.ie; soneill@mater.ie; research@stjames.ie; svhgethics@ucd.ie; crec@ucc.ie; joanne.oconnor@hse.ie. Should approval be granted, the corresponding author is happy to facilitate access in circumstances where data are fully and irrevocably anonymised, where data are being accessed for the purposes of further research and where a data access agreement is signed that meets any and all requirements specified by the Lead University and Principal Investigator.

### Extended data

Open Science Framework: Ryan K. National cross-sectional study of constipation in patients attending cancer centres. https://doi.org/10.17605/OSF.IO/ZXDSK ^13^.

This project contains the following extended data:

- Strobe checklist Ryan National cross sectional study of constipation.pdf
- Survey Instruments Ryan National cross sectional study of constipation.pdf

Data are available under the terms of the Creative Commons Zero "No rights reserved" data waiver (CC0 1.0 Public domain dedication).
