# Supplementary material for: A national cross-sectional survey of constipation in patients attending cancer centres in Ireland
Source: HRB Open Res. 2022 Jun 14;4:113. Originally published 2021 Oct 15. [Version 2] doi: 10.12688/hrbopenres.13315.2 (PMC9582576; doi:10.12688/hrbopenres.13315.2)
Supplement: Peer review history

# Peer review: Reviewer response for version 2

**DOI:** 10.21956/hrbopenres.14824.r32934

**Published:** 2022-10-19

**Authors:** Young D. Chang

**Affiliations:** 1 Department of Supportive Care Medicine, Moffitt Cancer Center, Tampa, FL, USA

Thank you for inviting me to review your article. This is a national cross-sectional study with a direct survey among 491 patients attending multi-designated Irish cancer centers to assess prevalence, severity, and management efficacy of constipation in cancer patients. The study showed again high prevalence (67.6%) of constipation among cancer patients. Female and opioid use were associated with higher constipation related symptom burden, but not patients under the management of palliative care specialists although constipation is generally expected more common and serious with high complex symptom burden. Interestingly, higher symptom burden with high CAS scores, opioid use and palliative care specialists were associated with laxative use. It is suggesting that palliative care may be an efficient way of the management for complex cancer related symptoms.

 My impression was that it was well written and also straightforward study design. I would just suggest some minor issues. 

1. I know that you indicated what SPC stands for. But, in the sentence of " *being known to SPC services*" in result section. It would be better to add " Specialist palliative care (SPC) in the main manuscript to avoid confusion for readers.
2. I am curious what are the differences between 'prescribed' vs 'OTC' in terms of chosen agent, dosage etc. OR was much higher with prescribed + OTC than prescribed and OTC. 

Is the work clearly and accurately presented and does it cite the current literature?

Yes

If applicable, is the statistical analysis and its interpretation appropriate?

Yes

Are all the source data underlying the results available to ensure full reproducibility?

Yes

Is the study design appropriate and is the work technically sound?

Yes

Are the conclusions drawn adequately supported by the results?

Yes

Are sufficient details of methods and analysis provided to allow replication by others?

Yes

Reviewer Expertise:

Cancer related pain and fatigue

I confirm that I have read this submission and believe that I have an appropriate level of expertise to confirm that it is of an acceptable scientific standard.

# Peer review: Reviewer response for version 2

**DOI:** 10.21956/hrbopenres.14824.r32317

**Published:** 2022-06-21

**Authors:** Bridget Candy

**Affiliations:** 1 Marie Curie Palliative Care Research Department, Division of Psychiatry, University College London, London, UK

I am happy with the revisions made and have no new comments on the revised article version.

Is the work clearly and accurately presented and does it cite the current literature?

Partly

If applicable, is the statistical analysis and its interpretation appropriate?

I cannot comment. A qualified statistician is required.

Are all the source data underlying the results available to ensure full reproducibility?

No source data required

Is the study design appropriate and is the work technically sound?

Yes

Are the conclusions drawn adequately supported by the results?

Partly

Are sufficient details of methods and analysis provided to allow replication by others?

Yes

Reviewer Expertise:

I am a palliative care researcher who has undertaken research in the treatment of opioid induced constipation

I confirm that I have read this submission and believe that I have an appropriate level of expertise to confirm that it is of an acceptable scientific standard.

# Response

**Published:** 2022-06-21

**Authors:** Karen Ryan

**Affiliations:** University College Dublin, Ireland

Many thanks for taking the time to review the latest version of the manuscript and for the helpful commentary that improved the paper. We are delighted that you are satisfied with the amendments.

# Peer review: Reviewer response for version 1

**DOI:** 10.21956/hrbopenres.14496.r30749

**Published:** 2021-12-10

**Authors:** Rita Wickham

**Affiliations:** 1 Rush University College of Nursing, Chicago, IL, USA

This is a well-written, clinically useful paper that I will look forward to reading in its final, edited form. There are minor editorial issues in punctuation that an editor will easily fix. My suggestions for improving this research report center on the tools used and operational definitions, which affect results and discussion:

- The authors should include details of instruments. Demographic data includes more than age and gender and this is just a list. Need more information about the CAS and the Bristol Stool Chart (figure might be useful, given contention of under-assessment). For instance, the CAS is an easily understood patient-completed tool that evaluates eight potential manifestations of constipation rated on a 3-point scale (no problem, some problem, severe problem). It is scored ----- etc.. Psychometric data and info about use in other populations/translation would strengthen choice of tool (Doğan & Aktuğ (2017 ^1^), Woolery *et al. *(2006 ^2^), Dal Molin *et al. *(2012 ^3^)).
- Do the same for the Bristol Stool Chart (Amarenco G (2014 ^4^), Srinivas *et al. *(2019 ^5^)). These are just examples.
- You state you identified patients taking opioid analgesics step 2 & 3, but page 4 mentions only step 3. Which did you actually include? Although most clinicians may be familiar with the ladder concept, some may not be. It may be useful to include a brief modifier, i.e. step 2 (codeine-like) and step 3 (morphine-like).
- Another question I had was regarding what laxatives are OTC and which are prescription in Ireland. I don't know if these differ from my (and other) country.
- Table 1: Do you want to call them participants, patients, or patient participants? Consider different layout for this table, with columns for yes (n/percent), no (n /%), and missing. Would be easier to read.
- On page 5, report of CAS scores will make sense if information about the instrument is included in the earlier section.
- Page 7: include the actual correlation coefficient before the p value.
- I had a question about laxative use that you may or may not be able to address in the discussion section: do patients typically self-medicate with laxatives, or do health professionals recommend they start with OTC laxatives?
- The last sentence in the first paragraph of the Discussion is confusing. Consider more direct: The factors most commonly associated with constipation in this sample of cancer patients receiving palliative care were opioid use and female sex (has this been previously found?): And how about stressing enhanced systematic constipation assessment.

Is the work clearly and accurately presented and does it cite the current literature?

Partly

If applicable, is the statistical analysis and its interpretation appropriate?

Yes

Are all the source data underlying the results available to ensure full reproducibility?

Yes

Is the study design appropriate and is the work technically sound?

Yes

Are the conclusions drawn adequately supported by the results?

Yes

Are sufficient details of methods and analysis provided to allow replication by others?

No

Reviewer Expertise:

palliative care, symptom management

I confirm that I have read this submission and believe that I have an appropriate level of expertise to confirm that it is of an acceptable scientific standard, however I have significant reservations, as outlined above.

## References

[1] : Validity And Reliability Of The Turkish Version Of Constipation Assessment Scale In Nursing Students. *Euras J Fam Med* .2017;6(2) :72-76 Reference source

[2] : A constipation assessment scale for use in pediatric oncology. *J Pediatr Oncol Nurs* .23(2) : 10.1177/1043454205285874 65-74 10.1177/1043454205285874 16476780

[3] : Validity and reliability of the Italian Constipation Assessment Scale. *Int J Palliat Nurs* .2012;18(7) : 10.12968/ijpn.2012.18.7.321 321-5 10.12968/ijpn.2012.18.7.321 22885963

[4] : [Bristol Stool Chart: Prospective and monocentric study of "stools introspection" in healthy subjects]. *Prog Urol* .2014;24(11) : 10.1016/j.purol.2014.06.008 708-13 10.1016/j.purol.2014.06.008 25214452

[5] : A cross-sectional study of stool form (using Bristol stool chart) in an urban South Indian population. *JGH Open* .2019;3(6) : 10.1002/jgh3.12189 464-467 10.1002/jgh3.12189 31832545PMC6891024

# Peer review: Reviewer response for version 1

**DOI:** 10.21956/hrbopenres.14496.r30752

**Published:** 2021-12-06

**Authors:** Bridget Candy

**Affiliations:** 1 Marie Curie Palliative Care Research Department, Division of Psychiatry, University College London, London, UK

This national survey provides prevalence rate of constipation for people attending cancer centres in Ireland. It finds a high prevalence of constipation, and limited use of laxations. The authors recommend better prescribing supported by clinical guidelines. 

 To make the article suitable for publication I would recommend some tightening in the writing, there are some redundant words and lack of order/flow between sections. The authors interchange between palliative care and Palliative Care. The results presentation does not follow the order set out in methods, as in first prevalence and then severity and then management. If they follow this in the abstract, results and then in brief in the discussion it will make the paper clearer.

- **Abstract and introduction:** It would be expected for the introduction to include what was said in the abstract background but to say it in more detail. For example, why are prevalence estimates so wide (50 to 90%). Also to set this in context what is the prevalence of constipation in the general population.
- **Method: **
  - They use fairly old data – you need to mention this in the discussion. 
  - Helpful for readers to know more on the Bristol Stool Chart, as in number of items and what the items are.
  - I note there is limited/no data collection on opioid intake, cancer severity, and ethnicity/cultural difference. All these factors will effect constipation/how patient manages constipation.
- **Results:**
  - The authors need to give the reader some indication of how representative the 491 patients were of all such patients in Ireland. Also what about refusal rate?
  - Participant characteristics – you have not detailed whether prevalence differs between in and out-patients.
  - Table 1 – it is not that informative to give detail as stated on cancer centre recruitment. For instance, why is there a range of recruitment rates. Is it the case that the larger centres provided more participants? 
- **Discussion: **Is this the first national survey in Ireland or even elsewhere – if so say so. They refer to your findings echoing other studies, it would be helpful if they could say whether these studies were in Ireland or elsewhere.
- **Conclusion:** Add Ireland to the first sentence.

Is the work clearly and accurately presented and does it cite the current literature?

Partly

If applicable, is the statistical analysis and its interpretation appropriate?

I cannot comment. A qualified statistician is required.

Are all the source data underlying the results available to ensure full reproducibility?

No source data required

Is the study design appropriate and is the work technically sound?

Yes

Are the conclusions drawn adequately supported by the results?

Partly

Are sufficient details of methods and analysis provided to allow replication by others?

Yes

Reviewer Expertise:

I am a palliative care researcher who has undertaken research in the treatment of opioid induced constipation

I confirm that I have read this submission and believe that I have an appropriate level of expertise to confirm that it is of an acceptable scientific standard, however I have significant reservations, as outlined above.
